# Supplementary figures and images for: The transcriptome of HTLV-1-infected primary cells following reactivation reveals changes to host gene expression central to the proviral life cycle
Source: PLoS Pathog. 2023 Jul 31;19(7):e1011494. doi: 10.1371/journal.ppat.1011494 (PMC10431621; doi:10.1371/journal.ppat.1011494)

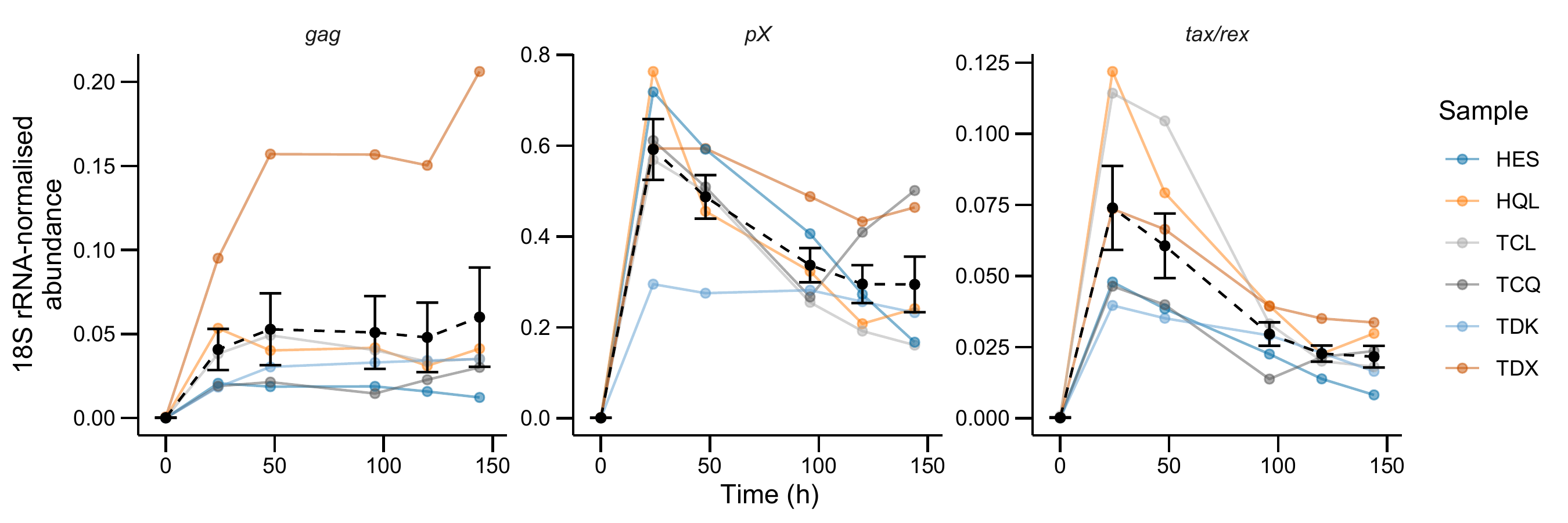

Supplement: S1 Fig — HTLV-1 sense-strand RNA trajectories during ex vivo culture, obtained using qRT-PCR.”gag” amplicons correspond to proviral region 2,017–2,203, whilst”pX” corresponds to region 8,000–8,161. tax/rex amplicons straddle the second exon junction. Coordinates presented for HTLV-1 sequence AB513134. Dark points and dotted lines represent mean ± SEM. (TIF) [file ppat.1011494.s001.tif]

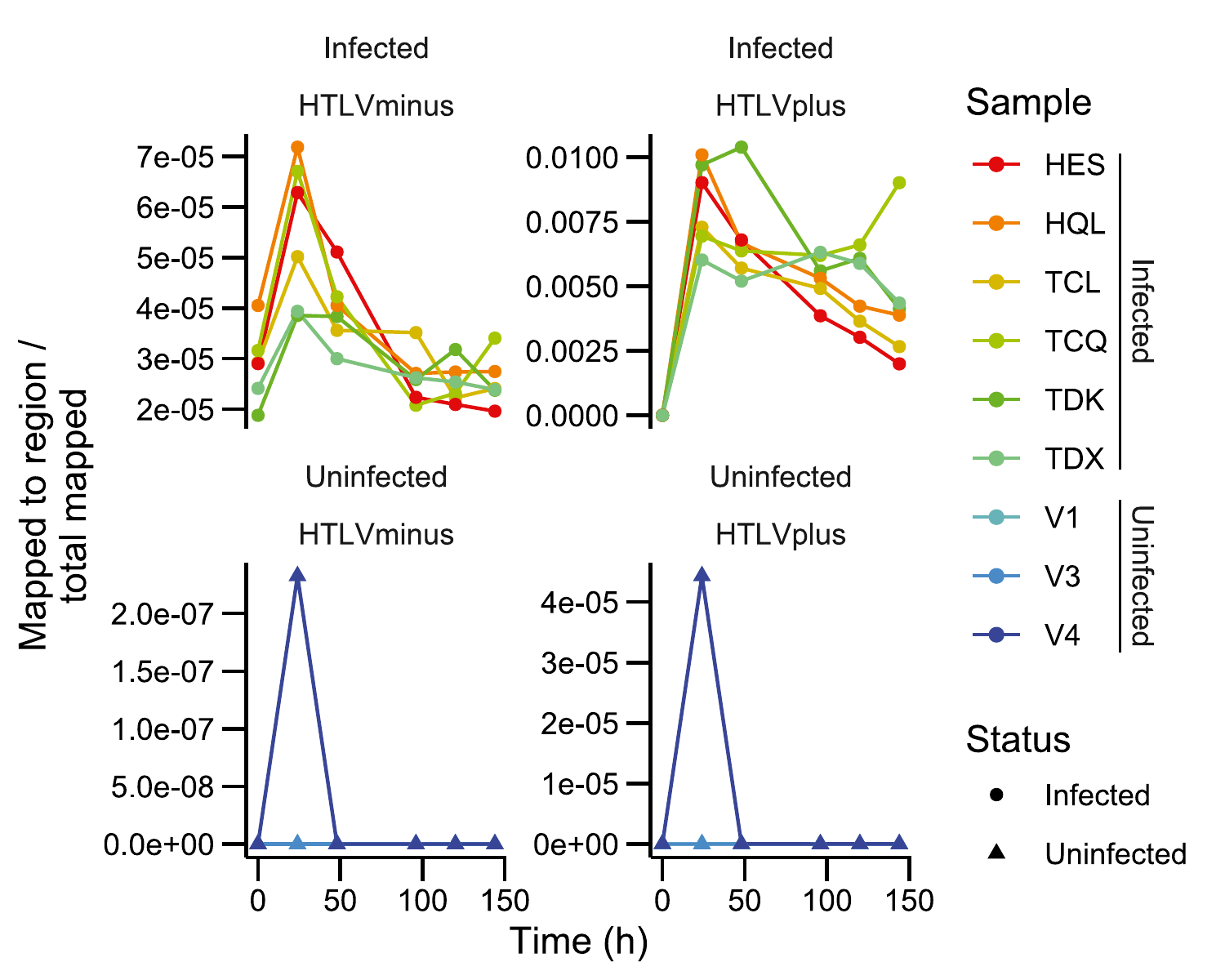

Supplement: S2 Fig — Values represent number reads mapped to HTLV sense or antisense strands normalised to the total number of reads mapped to hg38, with AB513134 appended as an additional chromosome, by STAR. (TIF) [file ppat.1011494.s002.tif]

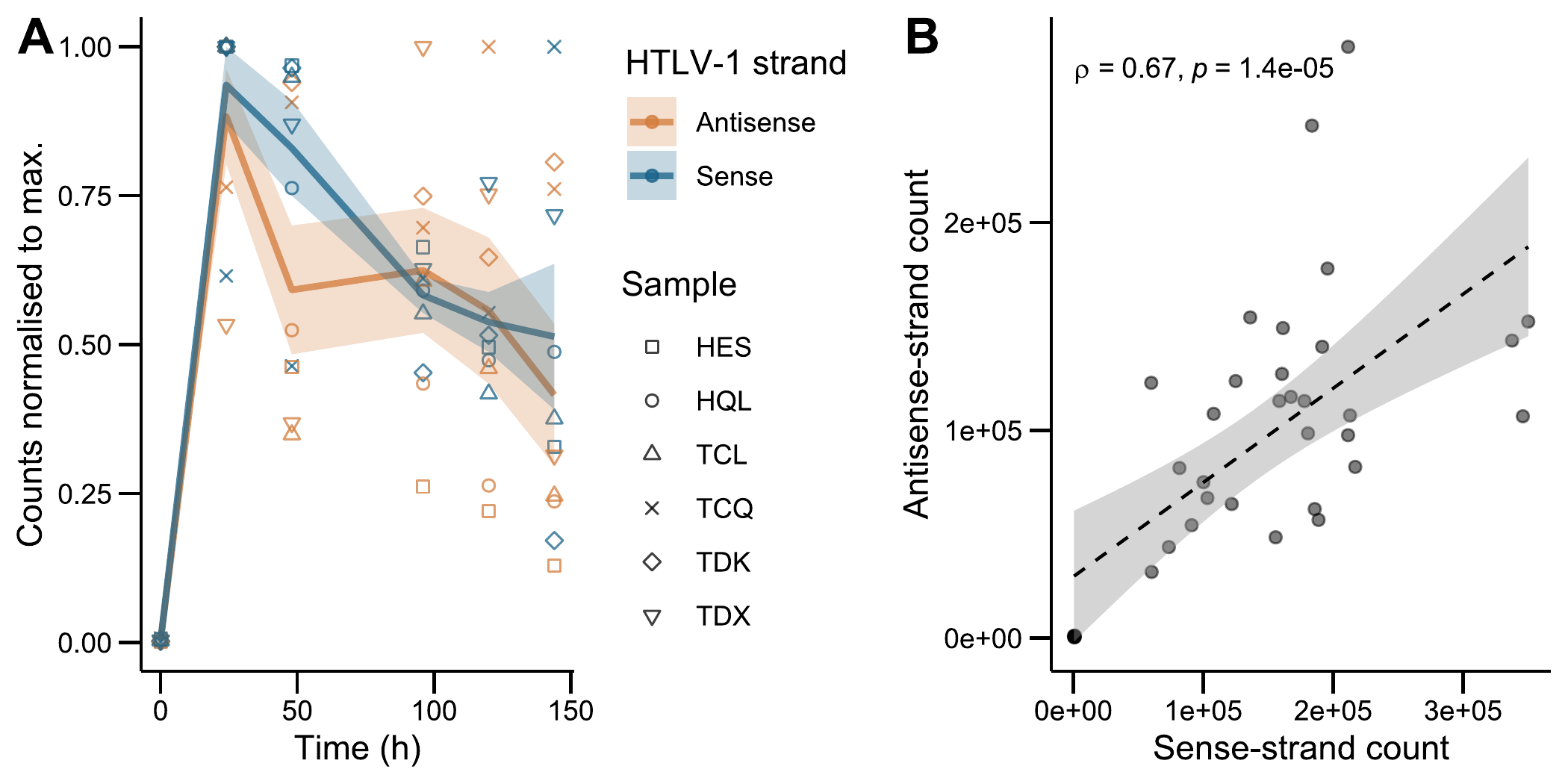

Supplement: S3 Fig — (A) DESeq2-normalised counts for sense and antisense strand transcripts normalised to their maximum values. (B) DESeq2-normalised counts of reads aligned to the proviral sense or antisense-strand exons. Linear model fit with 95% confidence interval shown. Statistics shown from Spearman’s rank correlation test. (TIF) [file ppat.1011494.s003.tif]

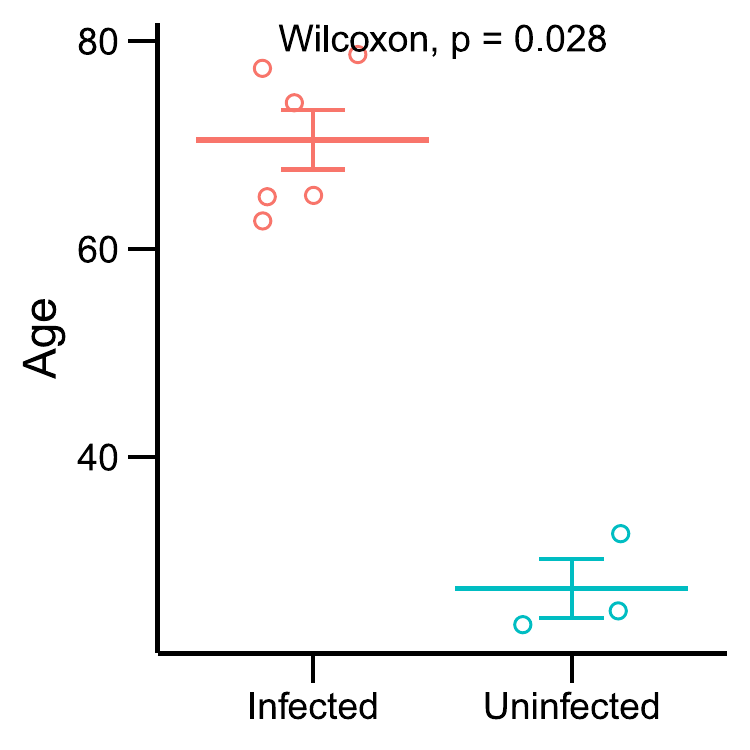

Supplement: S4 Fig — Uninfected controls were not age-matched with infected patients. p-value shown from Wilcoxon test. (TIF) [file ppat.1011494.s004.tif]

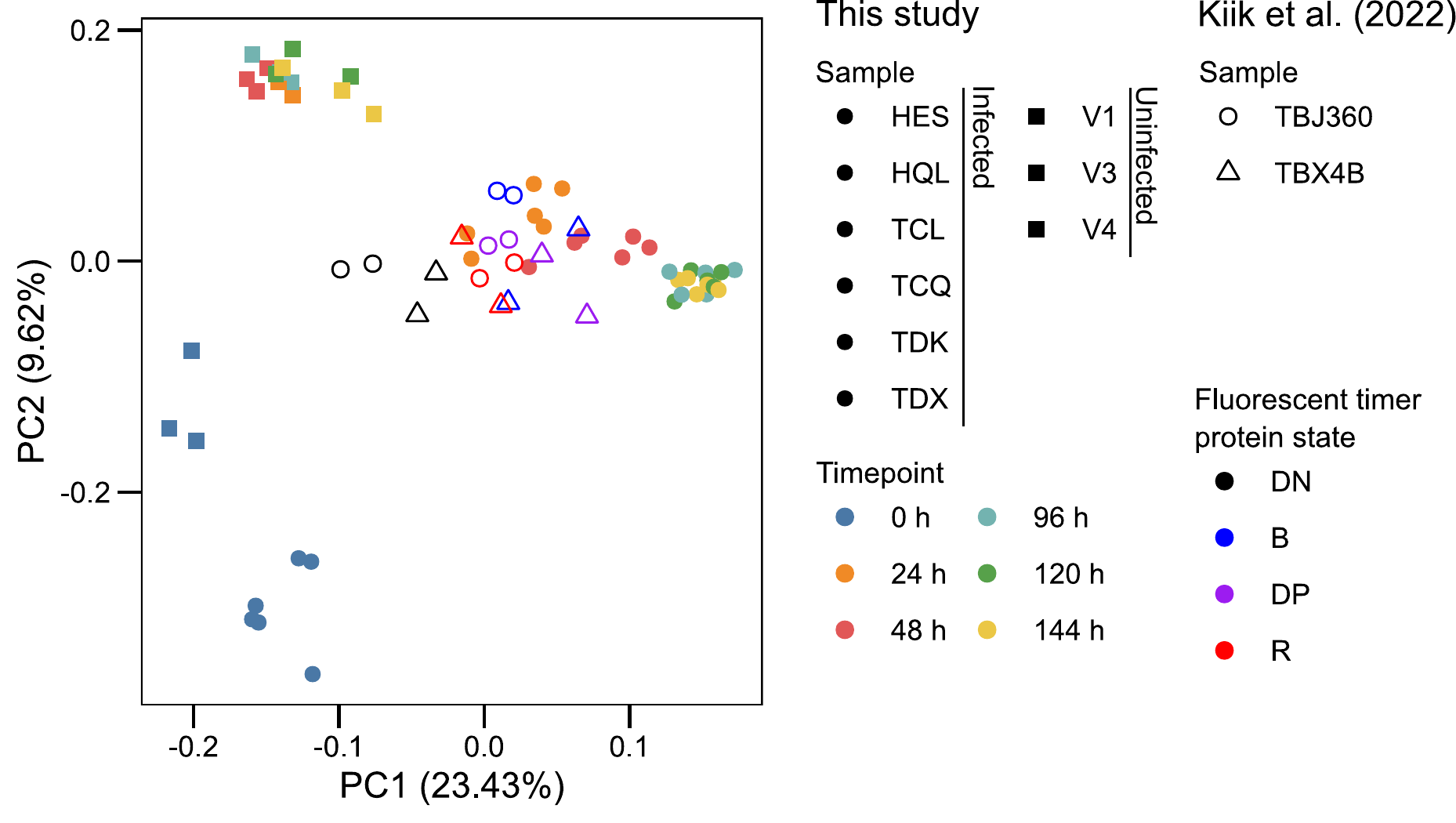

Supplement: S5 Fig — PCA analysis performed on VST-normalised data, subsequently z-scaled to correct for read count discrepancies between datasets. (TIF) [file ppat.1011494.s005.tif]

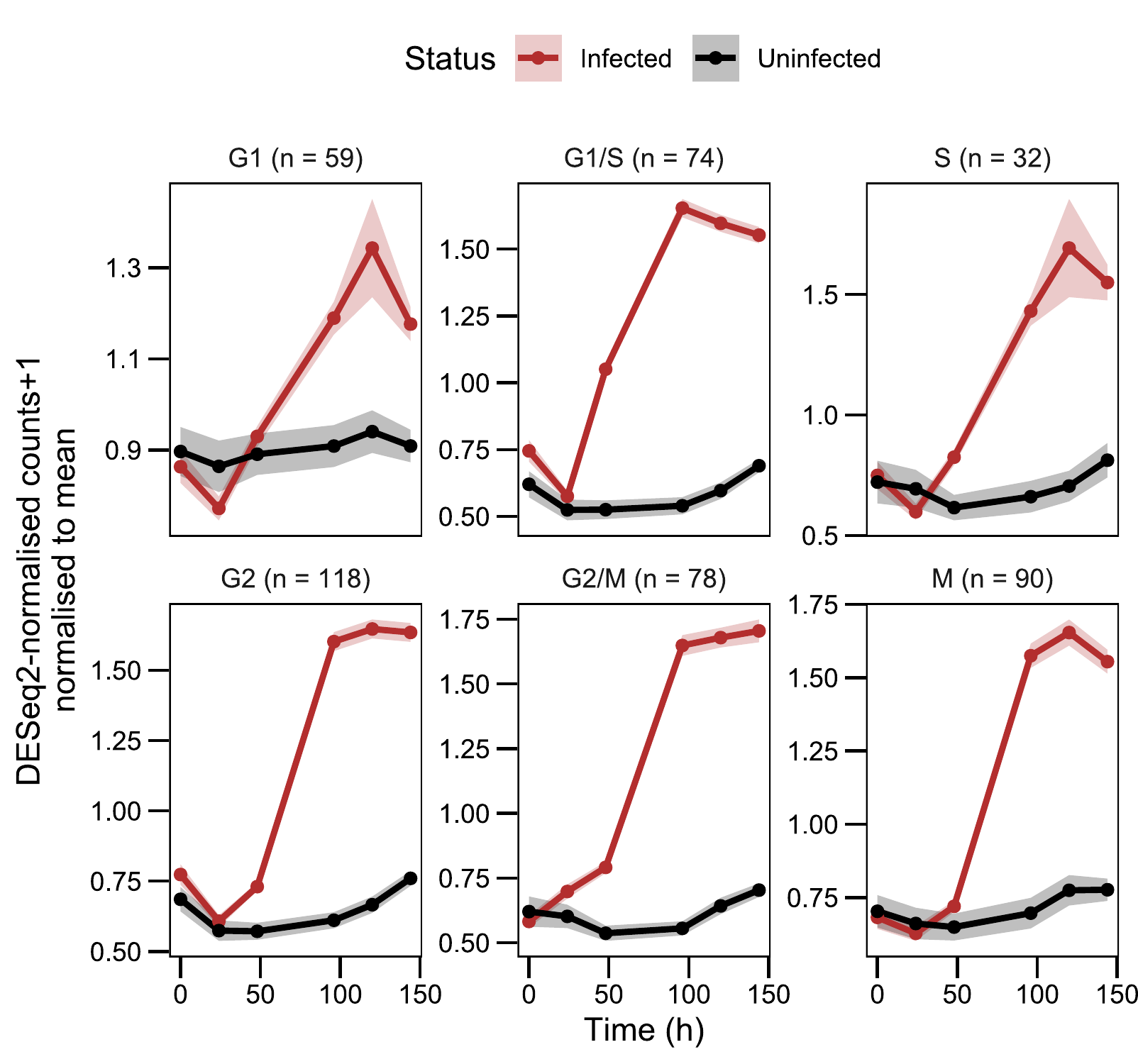

Supplement: S6 Fig — Mean trajectories of genes with peak expression levels at distinct cell cycle stages. Shaded regions represent ± SEM. (TIF) [file ppat.1011494.s006.tif]

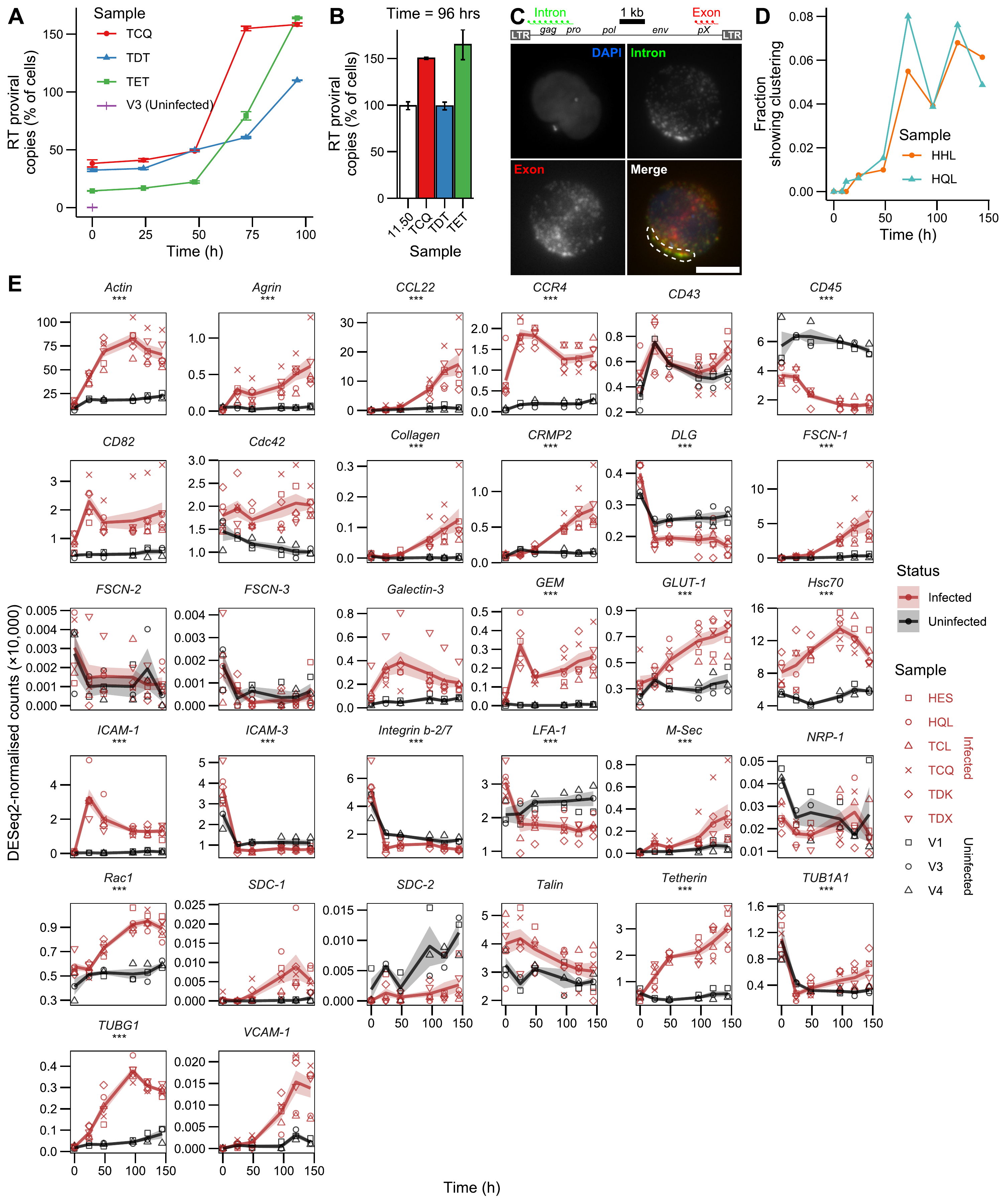

Supplement: S7 Fig — (A) Left: ddPCR PVL measurements for three infected samples and one uninfected control (V3). Right: Repeat measurement of 96-hour infected-cell samples, together with 11.50 positive control for 100% PVL. Error bars represent ± SD of two technical replicates consisting of sample dilutions. RT refers to reverse-transcribed. (B) Final timepoint from panel A, with 11.50 positive control for 100% PVL included. Error bars ±SEM. RT refers to reverse-transcribed. (C) Above: Schematic of HTLV-1 provirus and relative positioning of smFISH probes. Below: Example of cell showing clustering of unspliced proviral RNA near cytoplasmic periphery. Scale bar 5 μm. (D) Quantification of cells with visible clusters of unspliced RNA. (E) Trajectories of genes reported to influence horizontal infection. Asterisks indicate genes which change significantly over time and relative to uninfected cells. Summary lines and shaded areas represent mean ± SEM. (TIF) [file ppat.1011494.s007.tif]

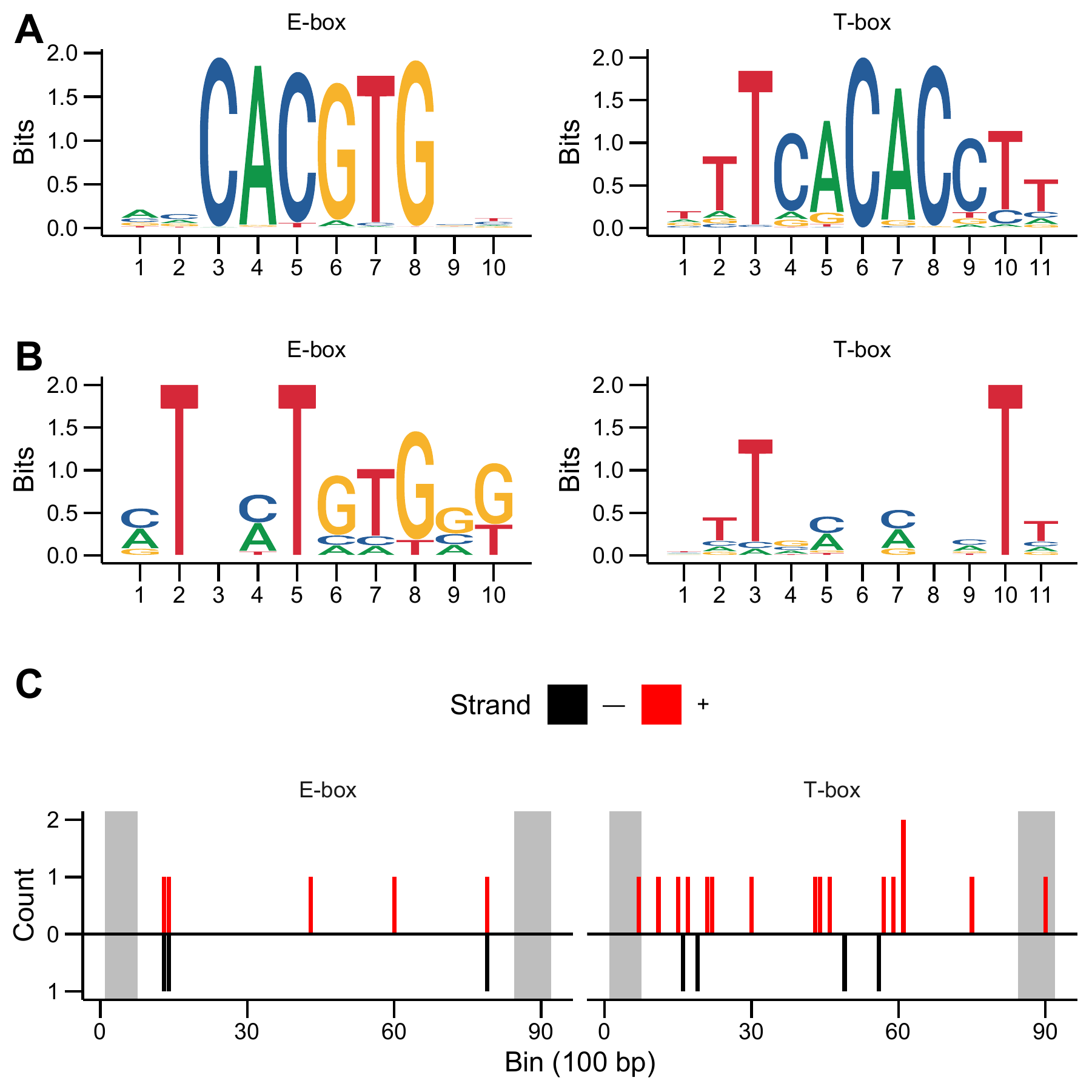

Supplement: S8 Fig — (A) Sequence logos of E- and T- box motifs obtained from JASPAR [110]. (B) Sequence logos of significantly matching (p < 0.001) sequences in HTLV. Generated using ggseqlogo [113]. (C) Distribution of sequences with significant similarity to E-box and T-box motifs along provirus. Grey boxes represent LTRs. (TIF) [file ppat.1011494.s008.tif]

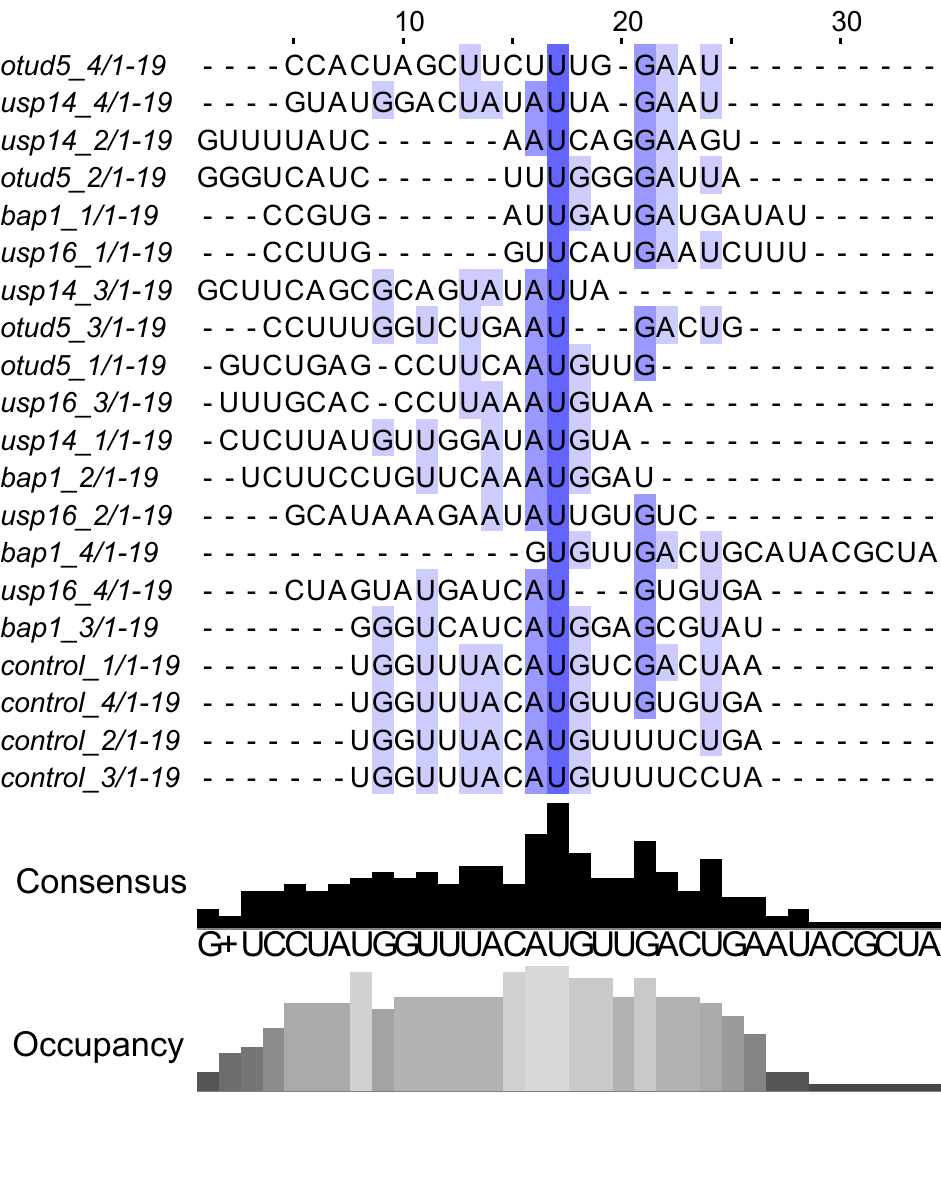

Supplement: S9 Fig — Sequences of siRNA fragments used to knockdown DUB transcripts, aligned using Clustal Omega [107]. (TIF) [file ppat.1011494.s009.tif]

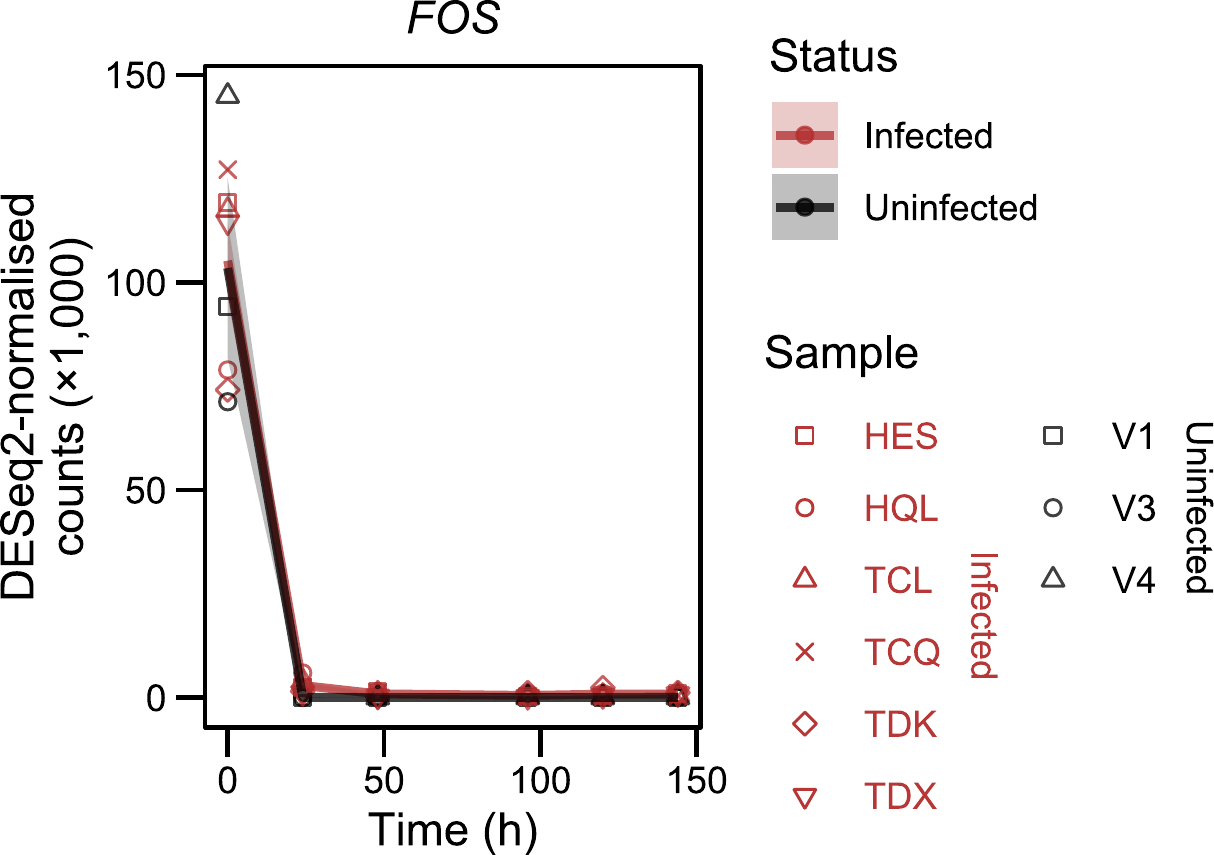

Supplement: S10 Fig — Lines and shaded areas represent mean ± SEM. (TIF) [file ppat.1011494.s010.tif]
