# Supplementary material for: The transcriptome of HTLV-1-infected primary cells following reactivation reveals changes to host gene expression central to the proviral life cycle
Source: PLoS Pathog. 2023 Jul 31;19(7):e1011494. doi: 10.1371/journal.ppat.1011494 (PMC10431621; doi:10.1371/journal.ppat.1011494)
Supplement: Attachment — Submitted filename: Aristodemou et al. - response to reviewers.docx [file ppat.1011494.s014.docx]

Aristodemou et al.: response to reviewers’ comments

Reviewer #1:

**1. Early activation of both sense and anti-sense transcription.**

We observed a significant correlation between anti-sense and sense-strand counts in figure S3, but this correlation does not of course prove that Tax directly regulates *HBZ* expression. We have amended the text (lines 187-190) to point out that our observations are consistent with a previous observation by Rende et al., 2011, and removed suggestions of direct regulation based on figure S3.

**Comparison with our previous results.** We recently (Kiik et al., 2022) reported an inverse correlation between the sense and antisense transcripts in one of two HTLV-1-infected T cell clones tested. The causes underlying the difference between these clones are still unclear. It was noted by Kiik et al. (2022) that *HBZ* followed the same trajectory as the transcription factor SP1 - our results for SP1 expression are consistent with this observation, as shown in data below, pulled from the trajectory viewer app:

**2. Regulation of sense and anti-sense transcription**

Given the observed correlation between transcription of the two strands and the identical effects of DUB inhibition on both strands, some degree of co-regulation is highly likely. The timepoints examined in this study are potentially too widely spaced to observe delays between upregulation of causal and dependent factors. However, we extend the discussion to include previous evidence on this (lines 633-643). Specifically:

Knockdown of *USP14*, *OTUD5* or *BAP1* also resulted in the reduction of *shbz* transcription. Together with the observed correlation between sense and antisense transcription, our results add to the evidence of overlap between the factors regulating expression from the proviral LTRs. High sense-strand transcript counts correlate with increased transcriptional bursting from the antisense promoter (Billman, Rueda, and Bangham, 2017), and the presence of Tax itself was found to increase expression from the 3´ LTR (Yoshida et al. 2008; Landry et al. 2009).

Our results differ in one respect from earlier work reporting an increase in *shbz* levels in CD8-depleted PBMCs from HTLV-1-infected individuals following 17-hour treatment with 50 µM PR-619 (Kulkarni et al. 2018). It is possible that this discrepancy is due to secondary or additional effects associated with the broad-spectrum DUB inhibition exerted by PR-619.

**3. siRNA inhibition of polycomb genes**

It would indeed be logical and interesting to directly downregulate some of the most interesting Polycomb Group genes through siRNA, in addition to examining whether H2AK119ub1 levels are inversely correlated with proviral expression during deactivation. However, this is beyond the scope of the present study.

Reviewer #2: Main points:

**1. Selection of subjects for study.**

HTLV-1-infected subjects were selected on the basis of their proviral load, which we required to be ~10% or higher, in order to extract a sufficient number of cells during the CADM1-sort for subsequent RNA extraction and sequencing. In addition to high proviral load, an oligoclonality index below 0.77 was a requirement for inclusion, to exclude leukemic samples with abnormal and non-representative expanded clones (cf. Rowan et al., 2016: PloS Pathogens 12(11): e1006030; Wolf et al., 2021: <https://pubmed.ncbi.nlm.nih.gov/33771974/>). An exception was made for sample HHL, which was used for smFISH of proviral transcripts but not for RNA-seq: this sample had an OCI > 0.77. An amendment has been made to the main text (lines 113-117) accordingly.

The sample from one asymptomatic patient (code HES) which did not display a signal by ddPCR was confirmed as HTLV-1-infected, as it was positive for HTLV-1 transcripts by RNA-seq and RT-qPCR, and clustered with remaining infected samples throughout the analysis.

**2. Further clinical details on HTLV-1-infected subjects**

In this study we focused on the differences between HTLV-1-infected and uninfected cells, and changes over time during ex vivo culture. Further specifically designed experiments would be needed to investigate possible differences between samples from ACs and from patients with HAM.

We present below data on the PVL and oligoclonality index measurements from the HTLV-1-infected individuals studied, taken within two years of the sample used.

| ID | Experiment | OCI-flow | PVL (% PBMCs) |
| --- | --- | --- | --- |
| HES | RNA-seq | 0.732 | 6.7 |
| HQL | RNA-seq/Imaging | 0.602 | 19.2 |
| TCL | RNA-seq | 0.735 | 9.7 |
| TCQ | RNA-seq | 0.702 | 24.5 |
| TDK | RNA-seq | 0.759 | 12 |
| TDX | RNA-seq | 0.701 | 7.7 |
| HHL | Imaging | 0.826 | 23.1 |
| TCQ | PVL timecourse | 0.702 | 24.5 |
| TDT | PVL timecourse | 0.754 | 3.3 |
| TET | PVL timecourse | 0.723 | 4.74 |

**3. Specifying clinical status of HTLV-1-infected and uninfected subjects.**

Legends and axis titles in figures 1, 2, 4, 5, 6, S1, S2, S3, S5, S7 and S10 have been updated, clearly indicating, in each instance, which samples are HTLV-1+ and which are uninfected controls.

**4. Please explain whether the removal of V4 t24 did or did not affect the analysis of differential gene expression by time course.**

The inclusion of an additional uninfected control sample could potentially alter the list of genes identified as differentially expressed. Nevertheless, all remaining uninfected samples cluster closely in the PCA, suggesting that changes brought about by the inclusion of an additional timepoint would be minor. Furthermore, since the sample was potentially cross-contaminated, we removed it from the analysis to avoid the introduction of doubt.

**5. Lanes 127 and 460-462, Fig S4: CADM1 expression on CD4+ T cells.**

This is an interesting point. CADM1 is not expressed on CD4+ T cells unless they are infected with HTLV-1. However, no study has addressed the question whether the frequency of induction of CADM1 expression on these cells varies with the subject’s age.

**6. Comparison of transcriptome data with Kiik et al. 2022.**

Transcript counts from Kiik et al. (2022) were re-quantified using Kallisto and the transcript index used in this study. VST-transformation was performed and the resulting data used together with VST-transformed transcript counts from our dataset to generate the PCA shown in figure S5. The legend in figure S5 has been updated to clarify which data points refer to which dataset.

The T cell clones used by Kiik et al. (2022) cluster together with HTLV-1-infected samples which have been in culture for ≥24 hours in the PCA, indicating similarity to uninfected samples and to infected samples prior to ex vivo culture.

**7. Use of raltegravir in cell culture**.

The integrase inhibitor raltegravir was not included in the experiment shown in figure S7A-B, as it was part of another series of experiments investigating horizontal transmission during ex vivo culture.

The smFISH data are from samples sorted for high CADM1 expression and cultured in the presence of raltegravir, in an identical manner to the samples used for sequencing.

We note the correlation between the ddPCR data in S7A-B and the imaging data in S7D, which suggests raltegravir does not impact the dynamics of transmission and reverse transcription of virions, at least during a single wave of infection.

To clarify these points, the methods sections relating to figure S7 and the horizontal infection experiments have been revised to clarify culture conditions in each experiment (Lines 813-823).

**8. Cell culture: How long raltegravir is effective on prevention of secondary HTLV-1 infection in the culture? Please describe if the sorted cells were cultured with or without any additional supplement up to 144 hours.**

Cells were cultured in the presence of 10 µM raltegravir, with the exception of the experiment shown in figure S7A-B. Raltegravir was included from the beginning of ex vivo culture, but not replenished over the course of the experiment, to avoid presenting the cells with additional stimuli.

Published work (Barski, Minnell and Maertens, 2019 - doi: [10.3389/fmicb.2019.01877](https://doi.org/10.3389%2Ffmicb.2019.01877)) suggests that even 2 µM of raltegravir is effective at preventing HTLV-1 integration over 16 days in culture, as raltegavir-treated cells presented 0.1% of the PVL measured in DMSO-treated controls. The source of infectious virus (MT-2 cells) was removed after 18 hours in Barski, Minnell and Maertens (2019).

The increase in reverse-transcribed proviral sequences observed also becomes apparent early in the timecourse, by 72 hours, regardless of the inclusion of raltegravir.

**9. Horizontal viral infection during ex vivo culture.**

The PVL was measured immediately after sorting to validate enrichment of infected cells, but repeated PVL assays were not made because of the small cell number. However, the samples used for smFISH analysis were sorted and cultured in a manner identical to the RNA-seq samples: therefore the accumulation of unspliced vRNA near the cell membrane over time likely also occurs in the RNA-seq samples.

**10. Lane 204-213: clarification of culture conditions.**

The methods section relating to the horizontal infection experiments presented in Fig. S7 has been revised (Lines 813-823) to clarify the culture conditions for each experiment. The smFISH samples were both sorted for CADM1^high^ and cultured in the presence of 10 µM raltegravir, in a manner identical to the RNA-seq samples.

**11. Tabulation of samples used in the study.**

All human samples used have been moved into a single table, table 1 (Line 704). The table also notes the experiments in which each sample was used. Additional information about clonal infected T cells used in the RNAi experiment is included in table 7 (Line 880).

**12. DUB trajectories in clonal cell lines; meaning of ‘serum.’**

It is not possible to obtain trajectories for clonal T cell lines, because (unlike the freshly isolated PBMCs) they are asynchronous with respect to proviral sense-strand expression.

‘Serum’ refers to fetal calf serum which was omitted from culture media during siRNA treatment, as per the manufacturer’s instructions. The manuscript has been updated to clarify this point (line 472).

**13. DUBs associated with proviral reactivation.**

It is not possible to determine with certainty from the present results which genes are directly involved in reactivation and which are simply co-regulated. The extensive overlap in function between the many (>50) DUB species frequently complicates the study of these enzymes. Proviral transcripts are reduced by approximately the same level, irrespective of the DUB knocked down. Below is the 96-hour timepoint data shown in figure 6D, replotted. Summary bars represent mean ±SEM.

**14. Lane 541: A link of a private website should not be included in discussion.**

The link to the data viewer has been moved to the data availability section in Materials and Methods (Line 909).

**Part III – Minor Issues**

Reviewer #1:

**1. Figure S7: technical details on horizontal transmission experiments.**

The methods section relating to figure S7 has been revised (lines 813-823) to clarify the culture conditions used in these experiments. Genomic DNA from the infected T cell clone 11.50 was included as a control for the accuracy of the ddPCR assay: this clone contains only one proviral copy per cell, and hence should produce a 1:1 ratio of proviral genome copies relative to cell numbers. This clone was not cultured like the other samples.

We speculate that the downregulation of LFA-1 may confer some protection in vivo, where the likelihood of encountering another actively transcribing cell is low. By contrast, the cells are brought into frequent close contact in these experiments, by settling at the bottom of a 5 mL FACS tube during culture.

**2. Would the infection process be expected to have a delay between the formation of unspliced proviral RNA clusters and the increase in RT proviral copies?**

There may indeed be such a delay, as the formation of unspliced proviral RNA clusters within host cells likely precedes the reverse transcription of vRNA following its entry into a target cell. However, the increase in RT proviral copy number appears to correlate well with the increase in unspliced proviral RNA clusters, so it is not certain from these data that there is any substantial delay.

**3. Author names are missing lane 585 and 670.**

Corrected.

Reviewer #2: Minor points:

**1. Materials and Methods – meaning of “RT”.**

Thank you for pointing out this potential confusion. All references to room temperature are now spelled out in full, and a note has been added to legend of figure S7.

**2 . Materials and Methods instrument used for cell sorting; cell purity measurement.**

A BD FACSAriaIII was used for sorting, and samples were run using the purity precision setting (yield mask 32/purity mask 32/phase mask 0). All samples were run using the same settings, with CST calibration run before sorting. To clarify these conditions and settings, an amendment has been made to the Materials and Methods section (lines 715-716).

Post-sort purity checks were not performed on all samples due to strict constraints on the numbers of cellc required for subsequent sequencing at multiple timepoints. We attach below post-sort purity data on the HTLV-1-infected sample TAT, which was ultimately not included in the study due to insufficient cell count. The purity of CADM1^high^ cells in this sample exceeded 96%.

**3. Materials and Methods (Lanes 585 and 670): Please remove the words “(authors?)”.**

Resolved (lines 722 and 820).

**4. Materials and Methods: There are multiple tables which were not referred in the manuscript.**

These tables are relevant to the directly preceding section; each table provides information on the methods described in that section. Each table is now referred to in the text.

**5. Symbols to denote statistical significance.**

The statistical methods used for each figure are specified in the respective figure legend. We follow the normal convention on using asterisks to identify statistically significant results, regardless of the test used.

**6. Lane 320: (Fig. 5E)?**

Resolved (Line 415).

**7. Lane 330: (Fig. 5F)?**

Resolved (Line 425).

**8. Lanes 335 and 337: (Fig. 5G)?**

Resolved (Line 431).

Reviewer #3: Below are some comments/suggestions to improve the quality of the paper.

1. **Age of subjects studied.**

In the text we draw the reader’s attention to the age difference between HTLV-1-infected subjects and uninfected controls. While we cannot formally resolve this point with the present data, we suggest that any differences in the expression of (for example) genes involved in epigenetic regulation are likely to be very small compared with the strong impact of HTLV-1 infection.

2. **Selection of CADM1+ cells.**

It is indeed possible that signalling may be altered by CADM1 selection; however, at present there is no effective alternative method to enrich for HTLV-1^+^ cells, which is necessary when using bulk sequencing methods. During ex vivo culture, CADM1 transcripts show a trajectory similar to that of proviral transcripts (see figure below), suggesting that even in cells where CADM1 is not expressed in vivo, it would likely become upregulated following proviral reactivation.

3. **The Authors might want to consider moving Figure S1 to the main manuscript, as it introduces the concept of the study.**

Figure 2A includes the same same trajectories for sense-strand transcripts based on the RNA-seq data as depicted in Figure S1, in which a fuller account of the individual transcripts is shown. The concept of the study is introduced in figure 1A.

4. **Legend to Figure S1, ‘pX’ sequences (8,000–8,161).**

This is correct: the pX sequence is included within all sense-strand transcripts. The data for figure S1 were obtained using a small subset of the RNA used for sequencing. The number of transcripts tested was therefore limited to tax/rex, gag and pX, which were used to investigate general sense-strand expression for each sample prior to commitment for sequencing.

Raw RNA-seq reads will be made publicly available once the manuscript is in its final form. Coverage should suffice for splice-isoform-specific quantification, which is currently beyond the authors’ expertise.

5. **Timing of peak tax/rex mRNA expression.**

Indeed, measurements earlier than 24 hours may have been interesting to observe. However, there is evidence in clonal HTLV-1-infected cell populations suggesting that tax/rex mRNA increases from 0-24 hours (<https://doi.org/10.1371/journal.ppat.1009577>; Fig. 5). The number of timepoints studied was limited by cell number and costs.

6. **Why was raltegravir added to the cultures?**

Raltegravir was included in the culture media to prevent the integration of additional proviruses following the reactivation of proviral transcription, which may result in additional waves of proviral transcription following initial deactivation further de-synchronising the samples in the study.
